# Integrated real-time imaging system, ‘IRIS’, Kangaroo feeding tube: a guide to placement and image interpretation

**DOI:** 10.1136/bmjgast-2021-000768

**Published:** 2021-10-28

**Authors:** Stephen Taylor, Kaylee Sayer, Danielle Milne, Jules Brown, Zeino Zeino

**Affiliations:** 1 Department of Nutrition and Dietetics, North Bristol NHS Trust, Bristol, UK; 2 Department of Anaesthetics, North Bristol NHS Trust, Bristol, UK; 3 Department of Gastroenterology, North Bristol NHS Trust, Bristol, UK

**Keywords:** enteral nutrition, nutritional support, endoscopic procedures

## Abstract

**Background:**

Lung complications occur in 0.5% of the millions of blind tube placements. This represents a major health burden. Use of a Kangaroo feeding tubes with an ‘integrated real-time imaging system’ (‘IRIS’ tube) may pre-empt such complications. We aimed to produce a preliminary operator guide to IRIS tube placement and interpretation of position.

**Methods:**

In a single centre, IRIS tubes were prospectively placed in intensive care unit patients. Characteristics of tube placement and visualised anatomy were recorded in each organ to produce a guide.

**Results:**

Of 45 patients having one tube placement, 3 were aborted due to refusal (n=1) or inability to enter the oesophagus (n=2). Of 43 tubes placed beyond 30 cm, 12 (28%) initially entered the respiratory tract but all were withdrawn before reaching the main carina. We identified anatomical markers for the nasal or oral cavity (97.8%), respiratory tract (100%), oesophagus (97.6%), stomach (100%) and intestine (100%). Organ differentiation was possible in 100%: trachea-oesophagus, oesophagus-stomach and stomach-intestine. Gastric tube position was confirmed by aspiration of fluid with a pH <4.0 and/ or X-ray. Trauma was avoided in 13.6% by identifying that the tube remained in the nasal lumen in the presence of a base of skull fracture (n=3) and in the stomach in the presence of recently bleeding polyps or mucosa (n=3). A systematic guide was produced from records of tube placement and interpretation of anatomical images.

**Conclusion:**

By permitting real-time confirmation of tube position, direct vision may reduce risk of lung complications. The preliminary operator guide requires validation in larger studies.

**Summary box**

**What is already known about this subject?**

- Tube misplacement is common but bedside guided tube placement can differentiate respiratory from gastrointestinal tract anatomy.

**What are the new findings?**

- From observations we developed a systematic, evidence-based operator guide to integrated real-time imaging system tube placement and identification of tube position using objective anatomical criteria.

**How might it impact on clinical practice in the foreseeable future?**

- The guide may facilitate operator training in use of a tube that permits early warning of tube misplacement to pre-empt complications.

## Introduction

An estimated 27 million nasogastric or orogastric and intestinal tubes are placed each year.^1^ End-of-procedure aspiration of fluid to check for a pH ≤5.5 or X-ray^2^ cannot prevent 0.5%, resulting in pneumothorax or pneumonia due to misplacement per se, despite tube removal before use.^3^ CO2 detection or X-ray can be done at a 30 cm or 40 cm tube depth, respectively. However, CO2 detection can fail^4^ and cannot warn of oesophageal misplacement, whereas a further X-ray adds delay to tube use.

In contrast, guided placement can potentially detect misplacement in real-time before damage is done, permit repositioning of the tube and confirming the tube position without delay. Fluoroscopy and endoscopy facilitate accurate placement but use is precluded by expense, risks from off-ward transport, invasive procedure, irradiation and delays to placement.^5^ Conversely, bedside ultrasound, electromagnet (EM) or direct vision-guided placement offers a solution.^6–8^ All require expert training but expertise in ultrasound may be the hardest to attain and requires a second operator. EM placement has a lower rate of undetected lung misplacement than blind placement in high-use centres (0.006% vs 0.01%), but it is higher than blind placement in low-use centres (0.35% vs 0.01%).^9–12^ Most errors were due to misinterpretation of the EM traces^13 14^ through insufficient training.^15^ In addition, interpretation based on manufacturer guidance may be inaccurate in about 25%–30% of cases.^11 16^ Finally, guided tube placement has been achieved by direct vision,^8 17^ However, there are insufficient data to determine safety and no evidence-based guide on which to train operators.

We documented a preliminary guide to integrated real-time imaging system (IRIS) tube placement from anatomical structures that were reliably visible.

## Methods

### Preparation

Cardinal Health trained author ST for 2 hours in the use of IRIS equipment, including during a tube placement. In addition, ST studied both Cardinal’s and online written and video training materials regarding IRIS and recognition of endoscopic images for approximately 8 hours. This included feedback on the accuracy of ST’s image interpretation by ZZ, a consultant gastroenterologist (endoscopist). To date, these materials do not provide a new operator with an adequately evidence based, systematic or comprehensive guide to IRIS tube placement.

### Equipment and tube placement

**Figure 1:**
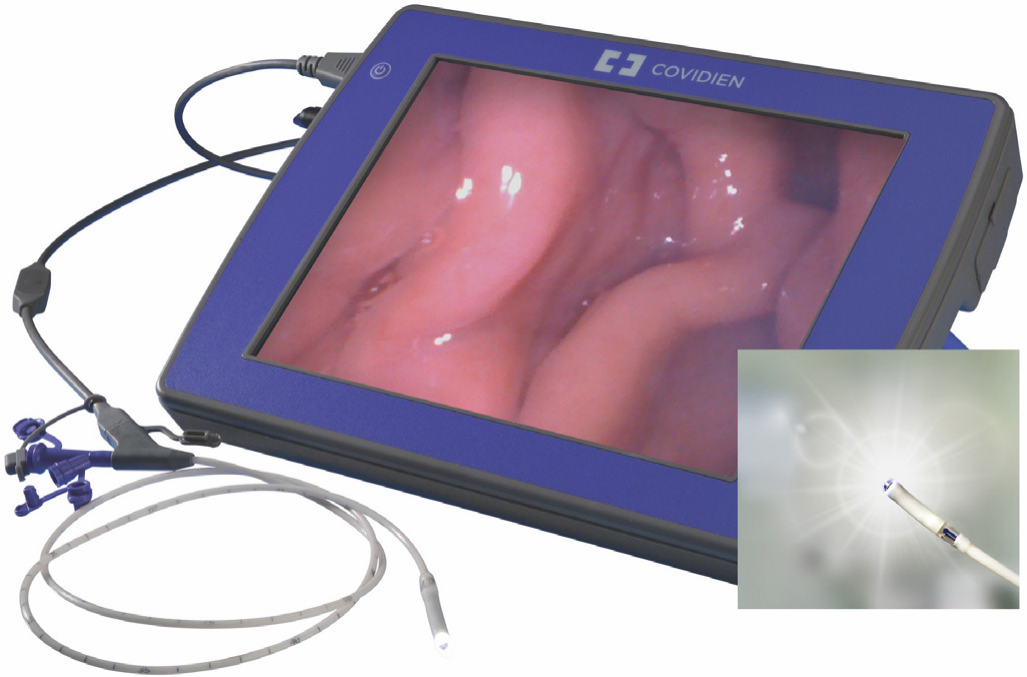
IRIS console, cable link and tube. IRIS, integrated real-time imaging system.

An IRIS tube incorporates a 3 mm camera within the tip to display an endoscope-like image via a cable link on a console (figure 1). Anatomical features can be identified in real time.

The external tube tip lubricant was activated in warm water before insertion via the nostril or mouth. A head tilt chin-down, jaw thrust manoeuvre or laryngoscopy were used, where necessary and safe to do so, to enable the tube to enter the oesophagus. Air insufflation and 5 cm tube retractions to clear the camera lens of mucus and slow tube advancement permitted recognition of anatomical features used to guide placement. Images were saved at noted tube lengths. When possible, nasogastric (NG) tubes were advanced into duodenum part-1 then withdrawn into the stomach to maximise recognition of anatomy and chance of aspirating fluid for a pH test.

### Patients

The tube was placed in a convenience sample of 45 adults (≥18 years) who required gastric or intestinal tube placement. This includes the unpublished anatomical data of 15 patients from a previous study.^18^ Exclusion was based on contraindication to enteral tube placement: Moribund, surgery or trauma to the nose or upper gastrointestinal (GI) tract contraindicating safe tube placement and patient consent refusal. A neurosurgeon and intensive care unit (ICU) consultant assessed the risk: benefit of nasal versus oral tube passage in the presence of a base of skull fracture, where use of direct vision may reduce risk.

### Aim, objectives, data collection and analysis

The aim was to produce a preliminary guide to IRIS tube placement based on the objectives of describing: (1) problems and solutions to placement and (2) anatomy from captured images. These images were interpreted by a non-endoscopist (ST, research dietitian) and compared against interpretation by ZZ (consultant gastroenterologist) and standard pH (gastric threshold ≤4.0) or X-ray (JB—consultant intensivist) confirmation of tube position.^19^ Patient demography, clinical status and adverse events were recorded. Analysis was only intended to be descriptive but will inform future studies on how operators can best use the IRIS system.

### Statistics

Parameters were tested for normal distribution using the Shapiro-Wilk test using ‘R Studio V.1.4’. Because several parameters were not normally distributed, descriptive statistics are presented as median (IQR) and percentage (%). Wilcoxon ranksum and Fishers exact tests were used to test continuous and binary variables, respectively. A p value <0.05 was considered significant.

## Results

### Baseline characteristics

One tube was placed by ST (n=44) or KS (n=1) in each of 45 patients (table 1). Most were medical patients, mechanically ventilated via an artificial airway and sedated.

**Table 1:** Patient demography, clinical state

Parameter	Detail	Median or n	*IQR or *%*
Number	(n)	45	–
Age	Years	58	50–73
Sex	Male	25	55.6
BMI	kg/m^2^	25	22.9–28.9
Height	cm	173.5	165–180
Weight	kg	77.5	65.5–93.8
APACHE 2	Score	13	10–16
Disease	Medical	17	37.8
	Neurosurgical (non-trauma)	9	20
	Surgery (general)	9	20
	Trauma	10	22.2
Consciousness	Awake	15	33.3
	Sedated	30	66.7
Artificial airway	None	16	35.6
	ETT	28	62.2
	Tracheostomy	1	2.2

BMI, body mass index; ETT, endotracheal tube.

### Tube and position

Placement was done on a median of day 2 (IQR: 2–5), via the nose (n=43) or mouth (n=2) using 12 Fr, 109 cm (n=38) or 10 Fr, 140 cm (n=7) tubes. Twelve tubes initially entered the respiratory tract (28%). Four tubes were removed when they reached the nostril (n=1) due to patient refusal, pharynx (n=2) due to inability to enter the oesophagus and upper stomach due to failure to achieve intestinal placement (n=1). Of 41 tubes left in situ, 30 remained in the upper stomach and 11 were pulled back from duodenum part 1 into the lower stomach. Gastric fluid was aspirated from 33 of 41 tubes (73%) and had a pH of ≤4.0 in 22 (54%). Of the 33 aspirates, the use of proton-pump inhibitors (PPI) led to a higher gastric pH (6 (5.75–6.5)) compared with non-PPI use (2.5 [2–4], p<0.0001). Three tubes were inadvertently removed preconfirmation. Of the 38 used for feeding, all were confirmed to be within the GI tract by a pH of ≤4.0 and/ or an X-ray (84%). X-rayed tubes were at least in the upper stomach (63%), lower stomach (34%) or duodenum part 1 (3%). However, 41% of tubes will have been more distal because the tip was below the X-ray frame.

### Ease of placement

Placement of the large tube tip via the nose was relatively easy, but 68% of placements were difficult at the pharyngeal level, with most requiring a head tilt (86.7%) and/or jaw thrust (46.7%) manoeuvre to enter the oesophagus (Appendix: Section 3.1). Also because we did not water activate the tube’s internal lubricant, to ensure accurate pH assessment, manoeuvring the guide-wire and tube only succeeded in reaching the lower stomach and intestine in 34% of attempts.

### Anatomical identification (figure 2: detail in online supplemental appendix: section 3.2 and 4)

**Figure 2:**
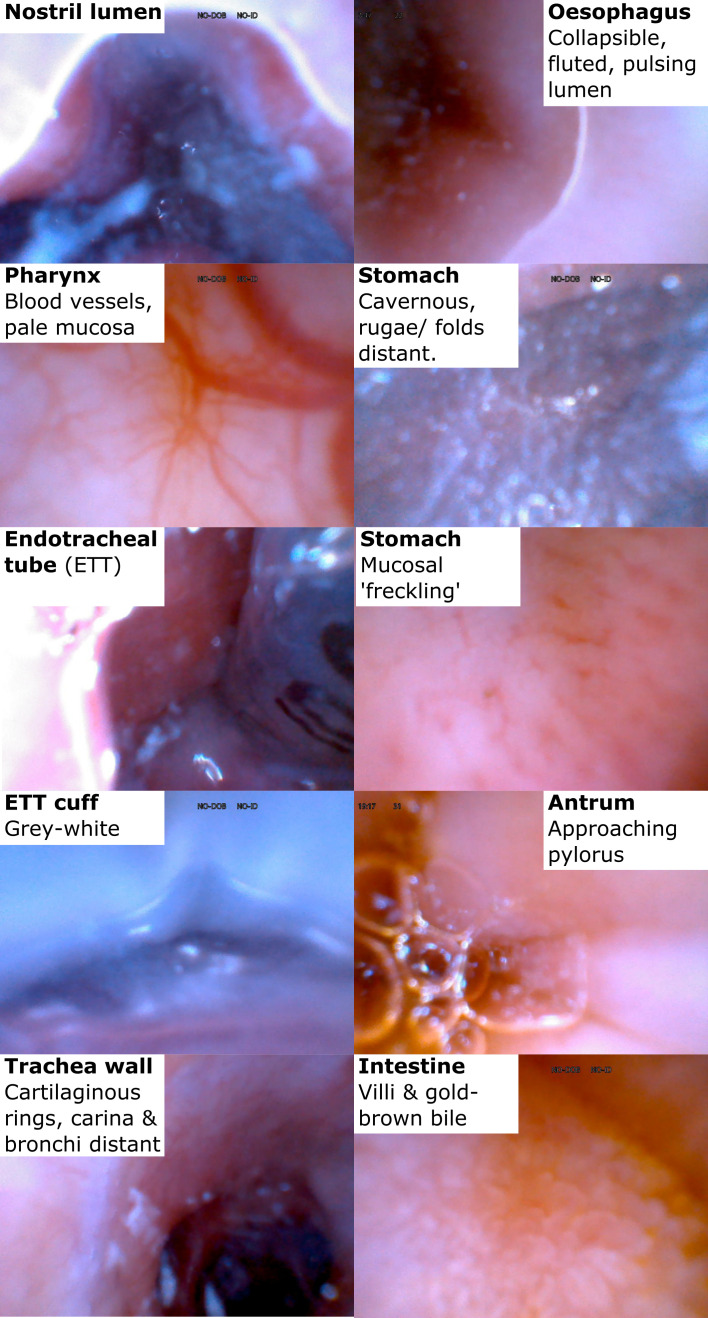
Interpretation of anatomical images seen using IRIS. IRIS, integrated real-time imaging system.

Identification of the oral or nostril cavities or oesophageal lumen (collapsible, fluted, pulsing mucosa) was possible in >97%, excepting one blood-filled nostril (online supplemental appendix 4.1) and one mucus-filled oesophagus (online supplemental appendix 4.4). However, we identified 100% of placements into respiratory tract (bronchi, carina, tracheal rings) (online supplemental appendix 4.3), stomach (cavernous space, folds/ rugae, freckle patterned mucosa) (online supplemental appendix 4.5) or intestine (villi) (online supplemental appendix 4.6). Differentiation was possible in 100% between trachea-oesophagus, oesophagus-stomach and stomach-intestine.

10.1136/bmjgast-2021-000768.supp1 Supplementary data

### Advantages and problems

There were specific advantages to IRIS tube guidance. The respiratory tract was avoided by manoeuvring the tube away from the identified airway in 6.8%. Tracheal placement was identified in 28%; one tube was removed and all the remainder repositioned in the GI tract. In addition, trauma was avoided in 13.6% by identifying that the tube remained in the nasal lumen in the presence of a base of skull fracture (n=3) and in the gastric lumen in the presence of recently bleeding polyps or mucosa (n=3).

In two patients, the tube luer became damaged and there was signal failure in one tube, resulting in removal, and one connector cable. The presence of an artificial airway in 9 (endotracheal tube; ETT, n=8; tracheostomy, n=1) of 12 tracheal misplacements was not significantly more than in those without an artificial airway (32% vs 20%, p=0.5). However, an artificial airway was associated with a trend to greater difficulty entering the oesophagus (76% vs 53%, p=0.17). So, though a similar proportion of patients with an artificial airway needed a head tilt manoeuvre (62% vs 53%, p=0.74), more of these patients needed a jaw thrust manoeuvre (48% vs 0%, p=0.001) and could not be done in one of two patients whose placement failed due to spinal injury. The common requirement for a head tilt or jaw thrust may preclude use of an IRIS tube, where these manoeuvres cannot be performed. Swallowing was requested in those patients without artificial airways and conscious; this may reduce risk of tracheal placement. IRIS tube tip size (5.6 mm diameter) and its curvature pointing towards the epiglottis when it reaches the pharynx may predispose to tracheal placement. In later placements, the operator straightened the guide wire, while within the tube, to reduce the curvature, but numbers were insufficient to analyse the effect. There were no placement-related adverse events, but one patient who was having bradycardic episodes before and after tube placement experienced reversible bradycardia during the initial stage of placement.

## Discussion

### Main findings

Direct vision using the IRIS tube facilitated placement by enabling recognition of the nasal or oral cavity (97.8%), respiratory tract (100%), oesophagus (97.6%), stomach (100%) and intestine (100%) and differentiated 100% of the trachea-oesophagus, oesophagus-stomach and stomach-intestine. Importantly, respiratory placement was detected pre-main carina, thus reducing risk of trauma or bacterial contamination. By comparison, blind and EM-guided placement is associated with the tube tip being a median or 18 cm (IQR: 16–23) and 12 cm (IQR: 9–15) beyond the carina, respectively.^18^ Blind placement is associated with a ~0.5% risk of pneumothorax or pneumonia.^3 20 21^ In addition, because IRIS has the potential to confirm position in real time, this would obviate the 2.1-hour delay for X-ray or, where the tube is misplaced would permit immediate repositioning rather than a 4.8-hour delay using X-ray.^18^

It was found important to advance slowly, sometimes with air insufflation ±a short withdrawal to permit identification of anatomy, particularly when differentiating the oesophagus from trachea, both of which can be mucus filled above the ETT or tracheostomy cuff. A head tilt and/or jaw thrust manoeuvre was commonly needed for the tube to enter the oesophagus. The large tube tip and possibly tube curvature appear to result in more tubes initially deflecting into the trachea (28%), similar to the 20%–35% found in previous studies,^8 17^ but more than the 11.4% of smaller-tipped EM-guided tubes.^11^ However, IRIS tubes detect misplacement precarina and usually without inducing coughing. Conversely, blind and EM tube placements may enter more deeply into the lung and induce coughing that leads the tube being repositioned before the X-ray or EM trace can evidence respiratory placement. The latter methods may, therefore, underestimate misplacement but fail to prevent pneumothorax or pneumonia.^18^

### Guidance for IRIS placement

From the findings and images captured during placement, a preliminary operator guide was written (online supplemental appendix section 4).

### Nose, pharynx and respiratory tract

IRIS permits the operator to identify that the tube was safely within either the nose or oral cavity. Pharyngeal mucosa is pale and, close-up, contains blood vessels that blanch when impacted. A head tilt and/or jaw thrust manoeuvre may then be needed to facilitate tube advancement into the oesophagus. To allow early differentiation of the oesophagus from trachea, 10–30 mL of air insufflation ±5 cm tube retraction cleared the lens and gave a good view of the oesophagus that is expected to collapse, have fluted walls and pulse. Identification of blood vessels and the z-line was less common. Mucus above an ETT or tracheostomy cuff usually obscured the tracheal wall’s cartilaginous rings and the cuff that is a translucent grey and may be bubble filled. However, beyond the cuff the trachea, carina and bronchi were clearly identifiable and the tube was carefully withdrawn.

### Stomach to duodenum part 1

On gastric entry, air insufflation and tube insertion or retraction aided visualisation of the cavernous structure and rugae or folds at a distance or, close up, a freckle-patterned mucosa. Air insufflation and use of a flexible tube tip may have helped advancement, but use of any length of a flexible tip was difficult; use of a non-water-activated lubricant would facilitate guide wire manipulation and tube manoeuvres using a flexible tip. This limitation and possibly the long, stiff tube tip may explain the low success rate in reaching the lower stomach. However, once the tube reached the lower stomach, sometimes with the aid of further air insufflation, advancement to duodenum part 1 was always successful. The pylorus was mostly only observed on slow tube withdrawal back into the stomach, but duodenal villi were easily visible. Finally, there was some uncertainty between gastric and intestinal placement on two images, similar to previous findings.^8^

### Limitations, recommendations and conclusion

This was a small, single centre, ICU study using operators already experienced in other guided tube placement techniques and only one non-gastroenterologist and one gastroenterologist interpreting images; therefore, generalisability may be limited. However, it was possible for a non-endoscopist to accurately identify major features of the respiratory and alimentary tracts. These are described to guide future users. To improve use of the IRIS tube, we think that its tip must be reduced in diameter and length and the angle removed. This would facilitate passage through the nose, entry into the oesophagus and ability to traverse GI flexures. The guide wire needs non-water activated lubricant to both permit subsequent pH checks and ease withdrawal and replacement around bends, enabling the tube to collapse into the lower stomach or traverse flexures during advancement. The IRIS tube makes real-time guided tube placement possible. This should reduce the risk of lung damage during accidental respiratory placement and its use in non-ventilated patients suggests that non-ICU use is applicable. Improvements to the tube should increase placement success rates. In the future, training material should include video of external placement alongside internal images and a photographic bank to extend operators' training experience.

## Data Availability

Data are available upon reasonable request.
