# Supplementary material for: Integrated real-time imaging system, ‘IRIS’, Kangaroo feeding tube: a guide to placement and image interpretation
Source: BMJ Open Gastroenterol. 2021 Oct 28;8(1):e000768. doi: 10.1136/bmjgast-2021-000768 (PMC8557303; doi:10.1136/bmjgast-2021-000768)
Supplement: Supplementary data [file bmjgast-2021-000768supp001.pdf]

IRIS Tube Placement and Interpretation: Preliminary Guide

Authorship: Stephen J Taylor, Kaylee Sayer, Danielle Milne, Jules Brown, Zeino Zeino.

Nutrition and Dietetics, Anaesthetics and Gastroenterology, Southmead Hospital, Bristol. UK.

Contents

Contents 1
1 Introduction 1
1.1 Aim 1
1.2 Objectives 1
1.2.1 Image interpretation 1
1.2.2 Tube and placement 1
2 Guidance 1
3 Background evidence 2
3.1 Ease of placement 2
3.2 Anatomical identification 3
4 Tube placement and image interpretation 5
4.1 Nose and mouth 5
4.2 Pharynx, epiglottis and endotracheal tube (ETT) 6
4.3 Respiratory tract 7
4.4 Oesophagus 8
4.5 Stomach 9
4.6 Intestine 10
5 Self-evaluation 11
5.1 Scenarios 11
5.2 Answers 13
6 Reference 13

1 Introduction

This is a preliminary guide based on the only study to systematically describe how to identify anatomy and thereby guide placement [Taylor et al, 2021]. This included 45 patients from one institution. It will undergo validation in future studies.

1.1 Aim

To enable IRIS tube operators to interpret screen image and thereby facilitate safe guided-tube placement.

1.2 Objectives

1.2.1 Image interpretation

Describe the sequence, characteristics and display images of specific anatomical points.

1.2.2 Tube and placement

Detail problems and solutions likely to be met during placement.

2 Guidance

This guidance is for adults (>18 years). Each anatomical area is described beside referenced images. Note that 'tube depth' *per se,* cannot be taken to indicate a specific anatomy due to differences in patient size, coiling within the GI tract and the possibility of being in the respiratory.

3 Background evidence

3.1 Ease of placement

Despite the large tube tip diameter, advancement through the nose (or mouth) was relatively easy. The patient who refused placement when the tip was 2cm into the nostril declined other treatments including pain relief. Lignocaine gel was applied pre-emptively to the noses of 5 conscious patients, to reduce discomfort, but it may also have reduced nasal swelling and facilitated tube passage (Table 4). However, 68% of placements were difficult at the pharyngeal level, predominantly in those with an artificial airway, most of whom required a head tilt or jaw thrust manoeuvre for the tube to enter the oesophagus. Two tubes could not be advanced beyond the pharynx, including one in which laryngoscopic placement failed and eventually required an airway to be directed into the oesophagus, through which a finer tube was placed. The IRIS tube tip appears to impact the pharyngeal mucosa and deflect towards the trachea.

Table 4 Techniques used during placement.

Area	Difficult placement		Technique used to overcome difficulty		
	N	%		N	%
Nose	2	4.4	Lignocaine*	2	100
Pharynx	30	68.2	Head tilt	26	86.7
			Jaw thrust	14	46.7
			Laryngoscopy	1	3.3
Stomach_upper*	27	65.9	Air insufflation	20	74.1
			10cm flexible tip	20	74.1
			≥20cm flexible tip	5	18.5
Stomach_lower*	3	14.3	Air insufflation	3	100

*Given pre-emptively in 5 patients to reduce discomfort; difficulty in only 2.

For clinical reasons of instability or comfort no attempt was made to advance some tubes beyond the upper stomach in 15 patients. In the remainder it was attempted to reach duodenum part-1, to help confirmation of tube position. The tube was then withdrawn and left in the lower stomach which, compared to upper stomach, reduces risk of the tube or feed regurgitating into the oesophagus. When attempting to reach duodenum part-1, 34% succeeded. However, where there was a difficulty moving from upper to lower stomach only 22% succeeded. We used air insufflation and withdrawing the guide-wire 10cm to create a flexible tip in 74% of attempts to advance into the lower stomach, but half the attempts failed. Use of a 20-30cm flexible tip was only used in 18.5% of attempts but was abandoned because the tube's internal lubricant had not been activated and therefore the guide-wire could not be re-inserted. We purposely avoided water flush-activation of the lubricant because of national guidance [NHSI, 2016]. Guide-wires were wiped dry on removal and stored in their labelled original pack.

3.2 Anatomical identification

Identification of nasal or oral cavities and oesophagus (collapsible, fluted, pulsing mucosa) was possible in >97%. However, it was possible to identify 100% of placements into respiratory tract (bronchi, carina, tracheal rings), stomach (cavernous space, folds/ rugae, freckle patterned mucosa) or intestine (villi). Differentiation was possible in 100% between respiratory trace-oesophagus, oesophagus-stomach, stomach-intestine.

Identification was done at placement by ST (dietitian) and confirmed by ZZ (gastroenterologist) either at placement or from a recorded screen image. Differentiation between respiratory versus GI tract and oesophagus versus stomach were always possible (Table 2). However, when examining four views of the dietitian's identification the gastroenterologist thought: a) A 'retroflex view of the pylorus' was probably the superior flexure; b) An 'antral view' may have been duodenum part-2, although the tube had been retracted and should not have been long enough to reach the duodenum; c) When uncertain of tube position leading to withdrawal, the gastroenterologist glimpsed tracheal cartilage not initially recognised by the dietitian and d) The dietitian tentatively identified villi partially obscured by blood; the gastroenterologist confirmed this.

Except for one patient whose nostrils were blood filled prior to tube placement, the nasal or oral cavity was visible in all patients, including three with base of skull fracture. Mucus prevented visualisation of the pharyngeal mucosa in 3 patients and the airway (epiglottis, ETT) and early part of the trachea in most patients. However, when entering the respiratory tract, except when the ETT cuff was spotted first, the carina and bronchi were always observed in the distance, usually with the tracheal wall visible close to the tip. All respiratory misplacements were detected and the tube removed before reaching the main carina.

Table 2 Anatomical identification.

Area	n*	Cases identified: Characteristic	n	%
Nostril/ mouth	45	Cavity	44	97.8
Pharynx	44	Blood vessels blanch; pale mucosa	41	93.2
Airway	43	Endotracheal tube	3	7
		Epiglottis	3	7
Respiratory tract	12	All	12	100
		Carina and bronchi	10	83.3
		ETT cuff	3	25
		Tracheal wall	9	75
Oesophagus	42	All	41	97.6
		Blood vessels	6	14.3
		Collapsible	41	97.6
		Fluted	31	78.6
		Pulses	27	64.3
		Z-line	1	2.4
Stomach	42	All	42	100

		Cavernous	41	97.6
		Folds or rugae	39	92.9
		Freckle pattern mucosa	32	76.2
Intestine	11	All	11	100
		Pylorus	5	45.5
		Villi	11	100

*Tubes reaching point or in a parallel tract.

Except for one mucus-filled oesophagus, all were recognisable, mostly by the collapsible and/ or fluted and/ or pulsing appearance. Blood vessels were harder to identify and the Z-line was seen only in one patient by the gastroenterologist. A short (≤5cm) tube withdrawal ± 10-30mL air insufflation was usually employed to clear the lens of mucus and open the lumen. The stomach was always identifiable by these characteristics: Cavernous space > folds or rugae > gastric pits. Of the 11 tubes reaching the duodenum, the pylorus was seen in 46%, but almost always on withdrawal because entry occurred too quickly. Villi were always seen though in one case villi were only tentatively identified by the dietitian because of blood covered the mucosa.

Differentiation between the respiratory tract and oesophagus and oesophagus and stomach were always possible. However, there were 2 cases of uncertainty regarding differentiation between the stomach and intestine (Table 3).

Table 3 Accuracy of anatomical identification.

Confirmation	Anatomy	N	%
Differentiation	Respiratory tract vs GI	11	100
	Oesophagus vs stomach	42	100
	Stomach vs intestine	11*	100*

* 1 was a tentative identification by ST because it was obscured by blood; confirmed by the gastroenterologist (ZZ). Another was thought by the gastroenterologist to be duodenum part-2, not gastric, but the tube had been retracted making intestinal placement unlikely.

4 Tube placement and image interpretation

4.1 Nose and mouth

Summary

As the tube is advanced, it should remain within the oral or nasal lumen and not cause trauma. Steady pressure and a change of direction may be needed to move through the nose and, in awake patient, 'humming' facilitates movement from nose to pharynx.

Comment	Depth	Images
The tube should be inserted 1cm up into the nostril then raised to an angle of 90° to the face **(as shown)**. This directs the tube along the floor of the nostril, the widest and least sensitive channel. In an oral approach, direct the tube down the centre, taking care to prevent buckling in the large orifice.<br><br>The proximal nostril will contain hairs. Protrusions of nasal concha may be seen and cause obstruction [1-3]. Mucus is present throughout [4]. Rotate the tube or change its angle and gently attempt to advance.<br><br>The tube should be seen to remain in an, often darkened, orifice. Impaction against mucosa [5], 'red out' or new blood [6] require tube retraction and re-appraisal of the placement.<br><br>In cases of high patient sensitivity, nasal obstruction or bleeding, topical lignocaine (lidocaine) gel, 'sniffed' into the nostril, will reduce swelling, discomfort and bleeding risk.	0-10cm	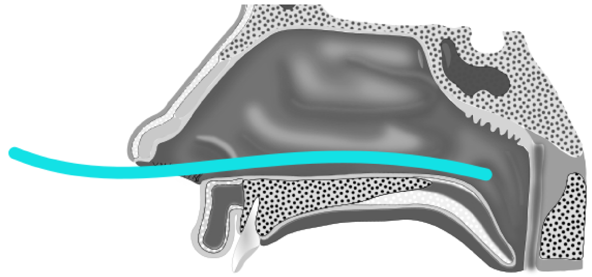<br>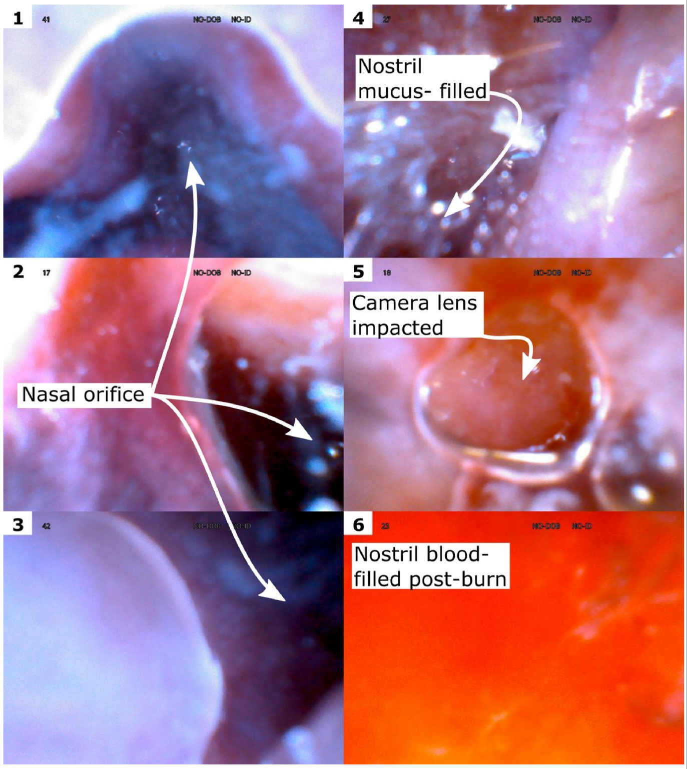

4.2 Pharynx, epiglottis and endotracheal tube (ETT)

Summary

The advancing tube commonly impacts and deflects off the posterior pharynx. Visualisation of the epiglottis or an ETT warns that it may be moving towards the trachea. Placing the head in a 'chin-down' position, a jaw-thrust manoeuvre, active swallowing and a slow advance facilitate tube movement into the oesophagus.

Comment	Depth	Images
The lens often passes through mucus and sputum at the confluence of the nose and mouth before the pharynx becomes visible. Pharyngeal mucosa appears pale pink with obvious blood vessels close-up [1] but these are often blurred. [2-3]. Tubes often impact the mucosa before advancing into the oesophagus or trachea.<br><br>Facilitate oesophageal placement, unless contraindicated, by tilting the head forward, chin down. A 'jaw thrust' is often more successful, because it moves the structures of the throat forward, making a channel for the tube to enter the oesophagus.<br><br>In very difficult cases, in sedated patients, an anaesthetist may place the tube into the oesophagus using a laryngoscope.<br><br>An endotracheal tube (ETT) is translucent grey with black numerals [4-5]. The epiglottis appears as a 'tongue' anteriorly, opening to the glottis, with the oesophagus collapsed, barely visible posteriorly [6]. Generally, if the ETT or epiglottis are visible the tube will need to be re-orientated, otherwise it will enter the trachea. This may necessitate removing the guide-wire and putting a 30° bend on it, 2-3cm from the tip. The guide-wire is then re-inserted and rotated to move the tube tip away from the airway towards the oesophageal entrance. Visibility of a feeding tube already in situ and confirmed as GI is a path to follow.	10-25cm	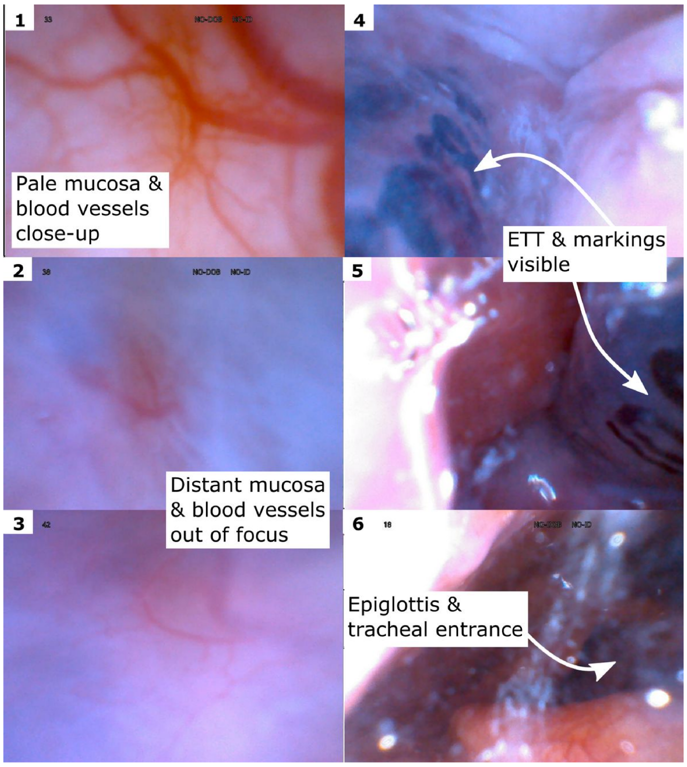

4.3 Respiratory tract

Summary

Tube entry into the trachea may be 'silent', not eliciting a cough, change in O2 saturation or a ventilator alarm. Visualisation of the vocal cords, ETT or tracheostomy cuff, cartilaginous tracheal rings, carina or bronchi should prompt immediate and careful tube withdrawal.

Comment	Depth	Images
During respiratory placement mucus, particularly that which collects above the cuff of an artificial airway, frequently blocks visibility. However, the ETT cuff may contain air bubbles [1], appear translucent [2] or grey-white [3]. Mucus may prevent visualisation of the cuff or respiratory anatomy when advancing between the ETT and the tracheal wall. But, beyond the cuff the respiratory tract is clearly visible:<br>■ Trachea: Rigid tube with cartilaginous rings [4].<br>■ Main carina: Point of Left-Right bifurcation [6].<br>■ Bronchi: Smaller rigid tubes, with sub-bronchi distally [5].<br><br>If any of the above features are seen or there are clinical signs of respiratory placement, slowly withdraw the tube to the pharynx. Assess whether it is safe to re-try.	>25cm	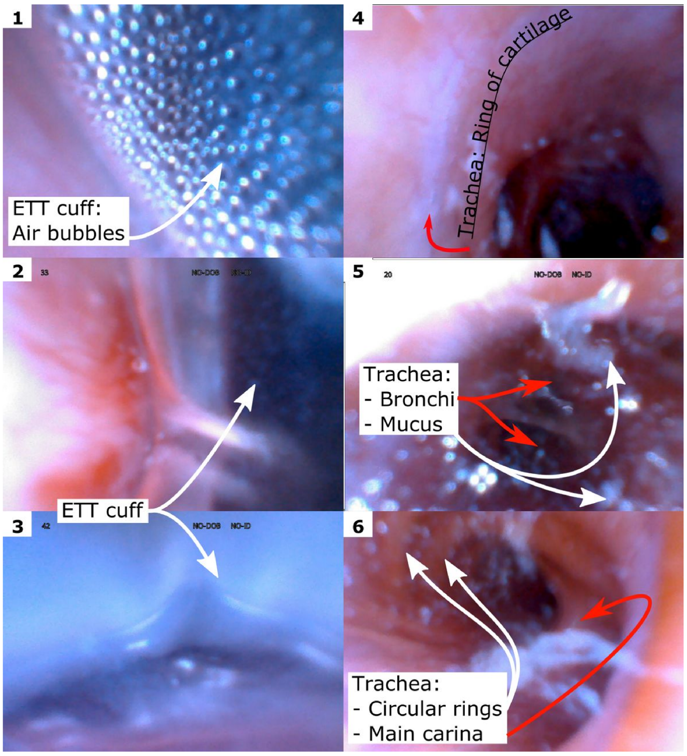

4.4 Oesophagus

Summary

Entry into the oesophagus is commonly obscured by mucus. Once entered, it is recognisable by its collapsible, fluted, pulsing wall allowing an easy advance. A 10-30mL air insufflation clears the lens, sometimes with a 5cm tube withdrawal. Only perform the latter when at least 10cm into the oesophagus to avoid returning to the pharynx.

Comment	Depth	Images
Mucus and the collapsed oesophageal walls usually obscure entry [1]. Food or feed debris may be visible [2]. Poor focus or soot (burn) blur features [3-4].<br><br>When oesophagus is collapsed and/ or the lens it may require a 10-30mL air insufflation ± 5cm tube retraction to open the view [5-6]. Avoid a long tube retraction that otherwise might pull the tube back into the pharynx, thus risk tracheal placement when re-advancing.<br><br>It should be possible to view the collapsible, fluted, pulsing oesophageal wall [7-8]. Blood vessels and the Z-line are harder to observe.	25-45cm	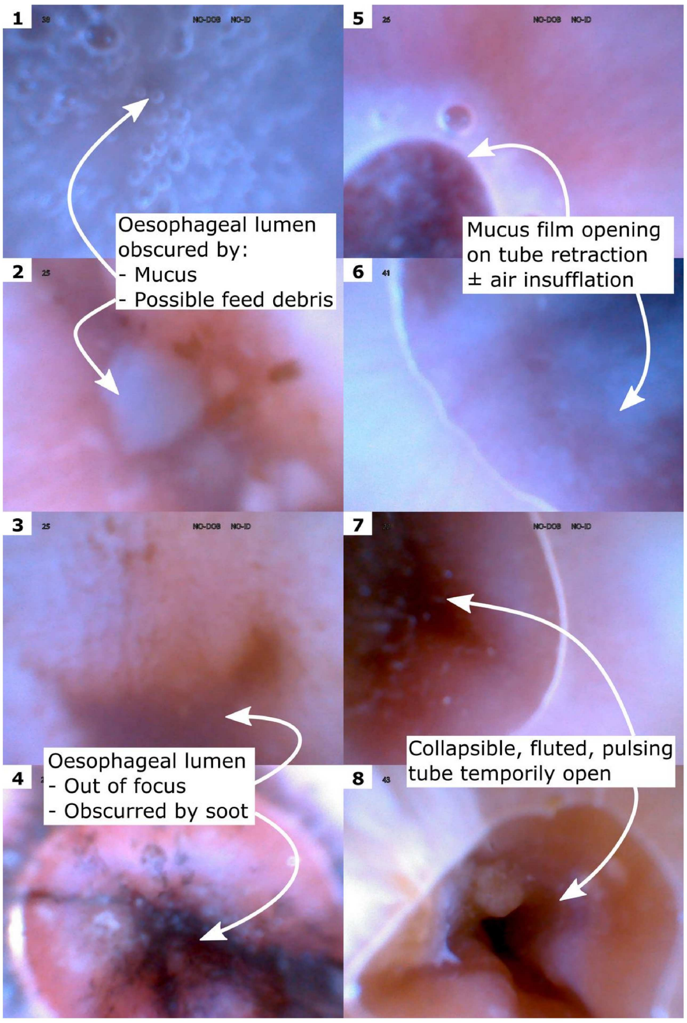

4.5 Stomach

Summary

Recognisable by the dark cavernous space, folds or rugae and, close-up, mucosal freckling. Failure of the tube to advance may require use of manoeuvres. Antral mucosa is smoother and paler and the pylorus may be briefly seen as an orifice before passing into duodenum part-1.

Comment	Depth	Images
The tube enters the stomach angled left and may quickly abut the greater curvature. If necessary, retract the tube a short distance. The most common gastric identifiers are that it is a cavernous (large) space [1-2] with folds and rugae distant [3] close-up [4]. Sometimes there is a cobbled or paved appearance [5-6]. Pulsing and peristaltic movements may be visible.<br><br>Close to the mucosa gastric pits may be visible [7-8]. Bile or feed or food debris may obscure vision [9-10].<br><br>Close to the antrum, the mucosa is pale [11] and smoother. Pyloric approach and entry may be masked by bile or mucus [12]. Pasages into the intestine may occur so quickly that the pylorus is only visualised on exiting the intestine.<br><br>During placement the tube may coil, retroflex, back towards the oesophagus. First, withdraw close to the oesophagus. A slow advance, air insufflation (250-500mL) and use of a flexible tube tip mitigate risk of coiling and encourage the tip to advance down the greater curvature.	45-75cm	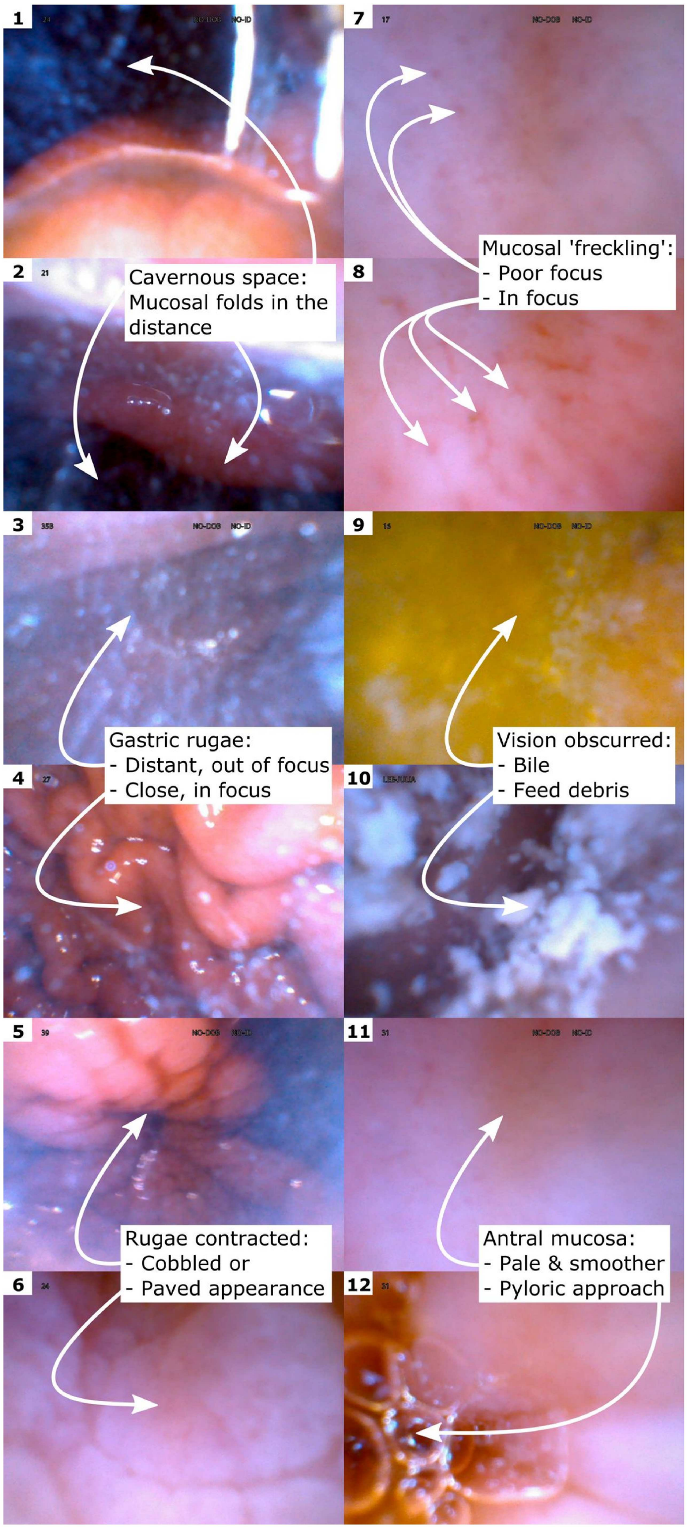

4.6 Intestine

Summary

Duodenal entry is sudden. Intestinal walls are close, covered in finger-like villi, often undergoing active peristalsis.

Comment	Depth	Images
The pylorus may be clearly seen when very slowly retracting the tube [1].<br><br>Bile appears yellow-brown [2]. Feed and food debris may be present.<br><br>Villi appear as pink-white finger-like projections [3-4]. They have a carpet-like appearance. Peristalsis may be visible.	>75cm	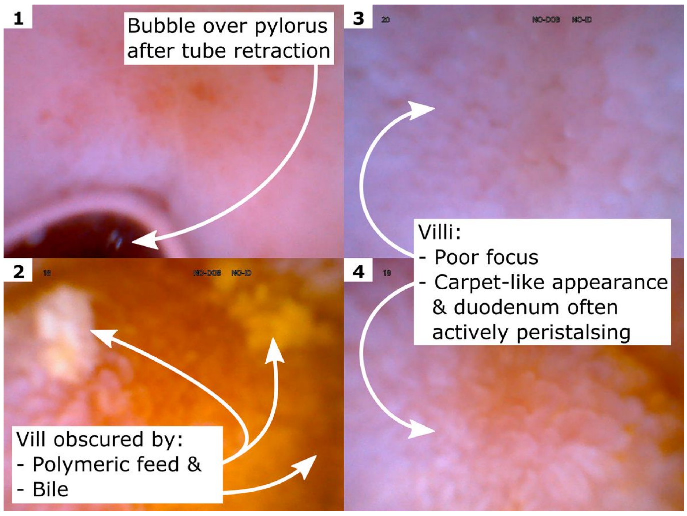

5 Self-evaluation

This section presents scenarios of tube placement and image interpretation:

1. Read the question, examine the images compared to those presented above & **note your answers.**
2. Open a new window showing 5.2 to view answers. Re-examine previous sections when necessary.

5.1 Scenarios

NB. Scenarios may be from different tube placements, not necessarily linked.

**Scenario**		**Image**
1	The tube has been inserted 5cm into the nostril.<br><br>What indication does this image give that the tube advance is safe?	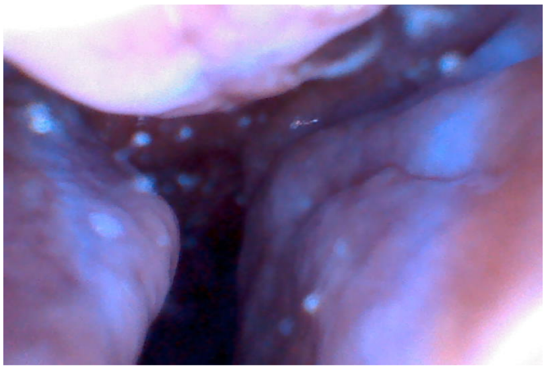
2	In a mechanically ventilated patient with an endotracheal tube in situ, 3 images are seen at distances from the nose:<br>• 18cm<br>• 25cm<br>• 30cm<br>There are no clinical signs or ventilator alarms to that the tube is misplaced.<br><br>a) Where is the tube at 30cm?<br>b) State what anatomy is seen and the characteristics used to identify it for each of the 3 images.	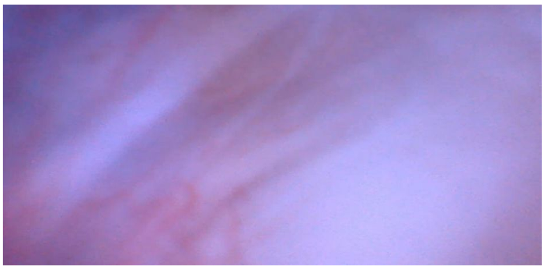<br>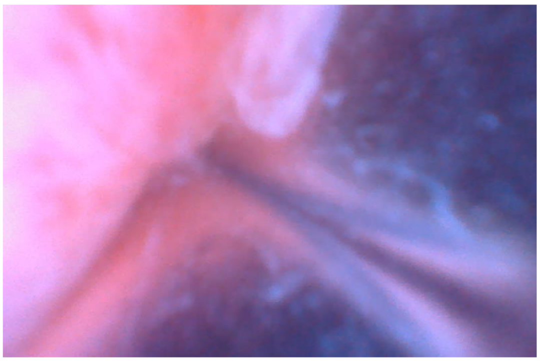<br>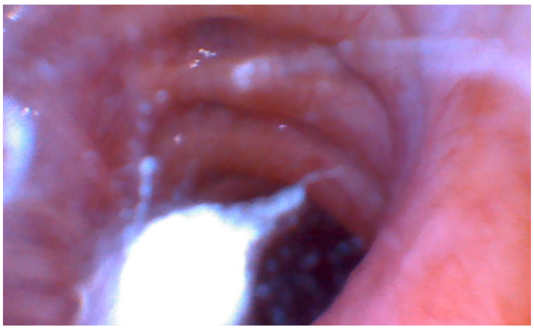

<table>
<tr><th colspan="2">Scenario</th><th>Image</th></tr>
<tr><td>3</td><td>Following scenario 2 the:
• NG tube was carefully withdrawn into the pharynx and
• Patient's head was tilted chin down and a jaw thrust manoeuvre done while slowly advancing the tube.

The following images were obtained.

a) Image 1 (29cm) shows mucus and no clear anatomy. If the IRIS lens does not clear spontaneously, how was the 2nd image obtained?
b) Identify the organ shown in image 2 (36cm)?
c) What characteristics can you identify that enabled you to answer b) and what characteristics might have been present when watching a continuous real-time placement?</td><td>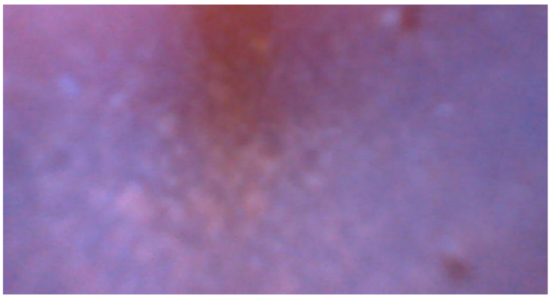
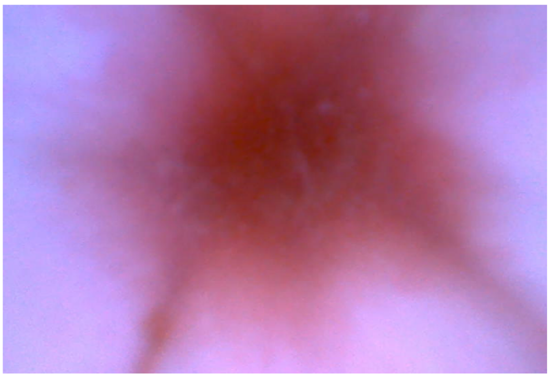</td></tr>
<tr><td>4</td><td>As the tube was advanced images were obtained at 60cm and 66cm.

a) Which organ is the tube in?
b) What 3 characteristics indicate this?</td><td>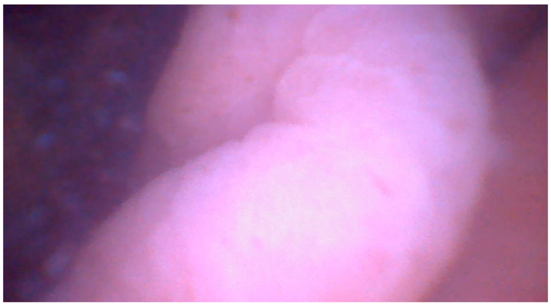
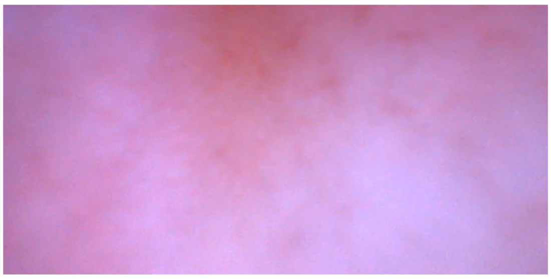</td></tr>
<tr><td>5</td><td>A tube advanced beyond 70cm from the nose, underwent a sudden advance and reached 80cm.

1. What techniques may help advance the tube from the upper to lower stomach?
2. Where is the tube in the image?
3. What characteristics indicate this?</td><td>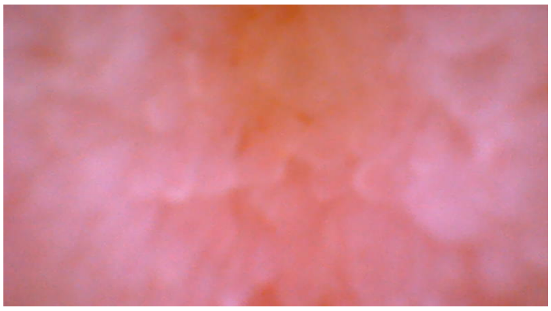</td></tr>
</table>

5.2 Answers

Q	Area	Answer
1	Nose	The tube is clearly in the nasal lumen (a darkened orifice). There is no blood or tube impaction against mucosa.
2	Pharynx and respiratory tract	a) Trachea<br>b):<br>i. Pharynx: Poorly focused, but the pale mucosa and visible blood vessels is typical.<br>ii. ETT cuff: The IRIS tube is passing the grey-white cuff; sometimes air bubbles are seen in the cuff fluid.<br>iii. The trachea has an open lumen and concentric cartilaginous rings. Distally it would show a carina and bronchi.
3	Oesophagus	a) A 10-30mL air insufflation, sometimes with a 5cm tube withdrawal clears the lens. Ensure the tube is 10cm into the oesophagus before withdrawing to avoid re-entering the pharynx.<br>b) Oesophagus<br>c) The lumen is fluted and in real-time collapses and pulses. It is not rigid and does not have cartilaginous rings.
4	Stomach	a) Stomach.<br>b) i. Gastric folds in a cavernous space. ii. A 'freckle' patterned mucosa.
5	Duodenum part-1	a) Slow advance, 250-500mL air insufflation (adult), 10cm guide-wire withdrawal to create a flexible tip. If the tube becomes retroflex, withdraw to remove the coil and repeat the advance.<br>b) Intestine. Because the tube has only just past the pylorus it will be duodenum part-1.<br>c) Finger-like villi and a smaller lumen. The gold-brown bile, partially obscuring the centre-screen, can also be seen in the stomach so does not help differentiate position.

Reference

NHSI. NHS Improvement. Nasogastric tube misplacement: Continuing risk of death and severe harm. Patient Safety Alert. 2016. NHS/PSA/RE/2016/006.

Taylor SJ, Sayer K, Milne D, Brown J, Zeino Z. Integrated real-time imaging system, 'IRIS', Kangaroo feeding tube: A guide to placement and image interpretation. BMJ Open Gastroenterology. 2021; : 1–5. doi: bmjgast-2021-000768
